# VAMS-Based Blood Capillary Sampling for Mass Spectrometry-Based Human Metabolomics Studies

**DOI:** 10.3390/metabo13020146

**Published:** 2023-01-18

**Authors:** Chiara Volani, Christa Malfertheiner, Giulia Caprioli, Søren Fjelstrup, Peter P. Pramstaller, Johannes Rainer, Giuseppe Paglia

**Affiliations:** 1 Institute for Biomedicine, Affiliated to the University of Lübeck, Eurac Research, 39100 Bolzano, Italy; 2 Interdisciplinary Nanoscience Center, Aarhus University, 8000 Aarhus, Denmark; 3 School of Medicine and Surgery, University of Milano-Bicocca, 20854 Vedano al Lambro, Italy

**Keywords:** VAMS, untargeted metabolomics, capillary blood, sample collection, mass spectrometry

## Abstract

Volumetric absorptive microsampling (VAMS) is a recently developed sample collection method that enables single-drop blood collection in a minimally invasive manner. Blood biomolecules can then be extracted and processed for analysis using several analytical platforms. The integration of VAMS with conventional mass spectrometry (MS)-based metabolomics approaches is an attractive solution for human studies representing a less-invasive procedure compared to phlebotomy with the additional potential for remote sample collection. However, as we recently demonstrated, VAMS samples require long-term storage at −80 °C. This study investigated the stability of VAMS samples during short-term storage and compared the metabolome obtained from capillary blood collected from the fingertip to those of plasma and venous blood from 22 healthy volunteers. Our results suggest that the blood metabolome collected by VAMS samples is stable at room temperature only for up to 6 h requiring subsequent storage at −80 °C to avoid significant changes in the metabolome. We also demonstrated that capillary blood provides better coverage of the metabolome compared to plasma enabling the analysis of several intracellular metabolites presented in red blood cells. Finally, this work demonstrates that with the appropriate pre-analytical protocol capillary blood can be successfully used for untargeted metabolomics studies.

## 1. Introduction

Conventional methods for health monitoring and disease diagnosis include the analysis of blood biochemical parameters. Generally, blood is collected through a venipuncture into different tubes that are then addressed to the central diagnostic laboratory for further analysis under appropriate storage and delivery conditions [1]. In recent years, great improvements in the analytical and detection techniques have allowed the number of samples required for the analysis to be reduced. This has opened the stage for the development of different microsampling techniques. Compared to an intravenous collection, where, usually, more than 1 mL is collected, microsampling approaches can capture even <100 µL of blood. The oldest microsampling technique is still very much used in clinics for the screening of newborn metabolic diseases and is called dried blood spot (DBS) [2]. This technique involves the use of a blood lancet and a sample collection card onto which the blood is spotted [2]. Because of the variations in sample quantification and the fluctuations of the hematocrit, groups have started working on the improvement of such technology. Volumetric absorptive microsampling (VAMS) is one of the recently developed technologies that has evolved from DBS [3,4], to allow single blood drop collection. In comparison to DBS, VAMS offers the advantage of overcoming issues related to the haematocrit (HCT), the volume of the blood spotted on the filter paper, and the sample homogeneity [3,4,5]. Additional microsampling technologies include the TAP capillary blood collection [6], the hemaPEN, and plasma microsampling [1]. An extensive description of these and other microsampling techniques is present in the review work of Lei and Prow [1]. 

In recent years, VAMS has become an effective tool and is used in several clinical applications, ranging from therapeutic drug monitoring [7,8] to proteomics [9] and metabolomics, as we recently described [10]. This microsampling technique foresees that blood is collected via a finger prick and becomes absorbed into a hydrophilic polymer [4]. Successively, the VAMS device is left to dry at room temperature and can be then stored for further analytical purposes [4,10]. VAMS sampling results in an easy, minimally invasive procedure that does not require venous access for blood withdrawal. As such, VAMS offers several benefits over conventional phlebotomy processes, and these, ultimately, can help to enable and improve precision medicine studies. Among them, VAMS facilitates participants’ self-collection of blood, hence supporting remote health monitoring and avoiding unnecessary extra hospital visits [9], which can be a burden, especially for the elderly. Moreover, it minimizes volume collection and does not, generally, require refrigerated transport and storage conditions [9,11].

Remote specimen collection improves patient compliance and participation in longitudinal studies. Population-based studies usually imply the enrollment of many participants; the CHRIS study is an example of this [12]. These studies often aim to investigate the genetic and molecular basis of age-related common chronic conditions and their interaction with the lifestyle and environment, so typically multiple sampling is expected.

Not only population studies can benefit from VAMS. As we recently learned during the COVID-19 pandemic, the need for social distancing to avoid unnecessary personal contact can represent a limitation for sampling that can be overcome by VAMS blood collection [13].

As mentioned above, after blood collection, the VAMS device can be stored for further analytical purposes. An additional advantage of this technology is that it has different applications; as we have recently shown, VAMS can be used to investigate the metabolomic profile of both animal models [14] and humans [10]. However, improper experimental design, sampling, handling, and storage conditions can strongly affect the consistency, reliability, and reproducibility of the data [15,16]. 

For this reason, it is extremely important to establish proper sampling, handling, and storage conditions prior to the specific sample analysis. Along this line, our previous paper aimed to investigate the pre-analytical strategies for the use of VAMS in human studies, including VAMS storage conditions and extraction procedures for metabolomic analysis [10].

The results suggested that, for long-term storage, VAMS devices should be stored at −80 °C after sampling to prevent changes in the overall metabolome profile [10]. However, the information on the short-term stability of the metabolome, as well as the differences of the metabolome sampled from capillary blood using VAMS versus peripheral venous blood, still remains to be clarified.

To close this gap, this study first investigates the short-term stability of VAMS samples. Secondly, we evaluated differences in the metabolic profiles between VAMS-sampled capillary blood to venous whole blood and venous whole blood-derived plasma samples (which is the default sample matrix for conventional studies involving human participants). To this end, we first stored VAMS samples for two weeks at different storage conditions and compared the resulting metabolomic profiles. To evaluate differences between the sample matrices, we designed a small study based on 22 healthy volunteers from whom we collected VAMS capillary and venous blood samples, as well as VAMS plasma samples and compared the resultant metabolite profiles.

## 2. Materials and Methods

### 2.1. Chemicals

All materials were obtained from Sigma-Aldrich (Sigma-Aldrich, Seelze, Germany) unless stated otherwise. Acetonitrile was purchased from VWR International (Radnor, PA, USA). Water was obtained from a Milli-Q water purification system equipped with an LC-Pak polisher (Merck, Darmstadt, Germany). All chemicals and solvents were of analytical grade or higher purity. Metabolomic amino acid mix standard containing isotopically labeled amino acids was purchased from Cambridge Isotope Laboratories (Cambridge Isotope Laboratories, Tewksbury, MA, USA).

### 2.2. Ethical Approval

This study was performed in accordance with ethical standards. The local ethics committee (Comitato etico del comprensorio sanitario di Bolzano, protocol number: 0100837-BZ) approved the study, and all participants provided written informed consent.

### 2.3. Blood Sampling and VAMS Storage Conditions

MITRA VAMS devices were purchased from Neoteryx (Neoteryx LLC, Torrance, CA, USA).

**Figure 1:**
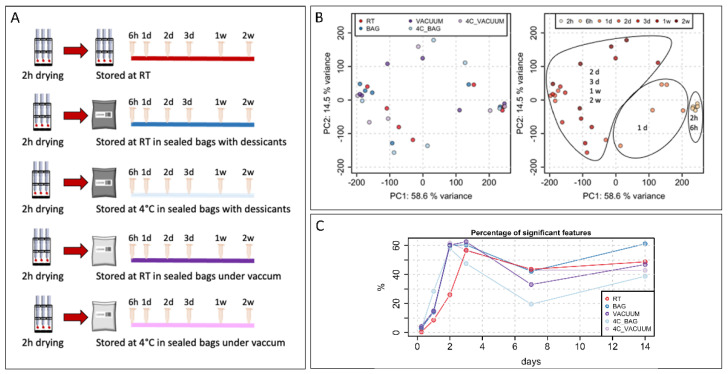
Short-term stability. (**A**) Experimental design to evaluate short-term stability of VAMS samples. (**B**) Principal component analysis (PCA) of all features after averaging technical replicates. Samples are colored by different experimental conditions or by time of storage. (**C**) Percentage of significant features for different storage conditions over time (h: hours; d: days).

(1) Short-term stability (Figure 1A): to investigate the short-term stability of VAMS samples, a pool of human blood samples was generated by pooling surplus EDTA blood samples from healthy subjects, which was obtained from the Transfusion Center of the Hospital of Bolzano. EDTA blood was selected to avoid the coagulation of native blood during VAMS collection.

One drop of the EDTA blood pool was adsorbed onto VAMS devices, which then followed the standard manufacturer’s procedure and were left to dry for 2 h at room temperature (RT). Next, VAMS devices were stored for different time points (2 h, 6 h, 1 day, 2 days, 3 days, 1 week, and 2 weeks) as follows:

− At RT in a protective outer casing;
− At RT in sealed bags with desiccants;
− At 4 °C in sealed bags with desiccants;
− At RT in sealed bags under vacuum;
− At 4 °C in sealed bags under vacuum.
(2) The evaluation of different sample matrices (Figure 1B): to investigate the qualitative and quantitative differences in the metabolome sampled from capillary blood on VAMS versus peripheral venous blood, a small study recruiting 22 healthy volunteer subjects was designed. Capillary blood and EDTA venous blood were collected from 11 age-matched females and 11 male participants thanks to a professional nurse at the Transfusion Center of the Hospital of Bolzano. VAMS devices were used to sample capillary blood, venous blood, as well as plasma samples. Capillary blood was sampled directly after the finger prick. Venous blood was collected into EDTA tubes, from which the blood was adsorbed onto VAMS. Next, blood was centrifuged for 15 min at 1500× *g* at 4 °C to obtain plasma. Plasma was then also absorbed into VAMS devices. All VAMS devices were left to dry for 2 h at RT; then, they were stored at −80 °C prior to the extraction procedure.

### 2.4. Metabolite Extraction Procedure

For sample extraction, all VAMS devices were left to thaw for around 45 min. Mitra tips were then rehydrated with MilliQ water for 5 s, then moved to Eppendorf tubes containing 200 μL of the extraction solution acetonitrile/water (70:30, *v*/*v*). Samples were sonicated for 15 min at 20 °C and then vortexed at 1200 RPM for 60 min at RT. VAMS tips were removed, and the samples were centrifuged at 1800× *g* for 10 min at 20 °C to remove the protein-like material. The supernatant was then filtered using a Sirocco Positive Pressure-96 processor applying 12 psi pressure (Waters Corporation, Milford, MA, USA). The extract was next evaporated to dryness under a vacuum at 35 °C for 120 min in an EZ-2 vacuum evaporator (Genevac, Ipswich, UK). Right before the analysis, samples were reconstituted with 150 μL of acetonitrile/water (50:50, *v*/*v*) solution, containing the labeled amino acid standards at the following concentrations: alanine 13C315N (0.9 μg/mL), arginine 13C615N4 (1.8 μg/mL), aspartic acid 13C415N (1.3 μg/mL), cystine 13C615N2 (1.2 μg/mL), glutamic acid 13C515N (1.5 μg/mL), glycine 13C215N (0.8 μg/mL), histidine 13C615N3 (1.6 μg/mL), isoleucine 13C615N (1.3 μg/mL), leucine 13C615N (1.3 μg/mL), lysine 13C615N2 (1.5 μg/mL), methionine 13C515N (1.5 μg/mL), phenylalanine 13C915N (1.7 μg/mL), proline 13C515N (1.2 μg/mL), serine 13C315N (1.1 μg/mL), threonine 13C415N (1.2 μg/mL), tyrosine 13C915N (1.8 μg/mL), and valine 13C515N (1.2 μg/mL). Pooled QC samples were prepared by pooling together an equal amount (10 μL) from each extracted sample.

### 2.5. Mass Spectrometry-Based Metabolomics

All samples were analyzed using ultra-high-performance liquid chromatography (UHPLC) (Agilent 1290; Agilent Technologies, Santa Clara, CA, USA) coupled to a Q-TOF mass spectrometer (TripleTOF 5600+; AB Sciex, Foster City, CA, USA). The chromatographic separation was based on hydrophilic interaction liquid chromatography (HILIC) and performed using an Acquity BEH amide, 100 × 2.1 mm column (Waters Corporation, Milford, MA, USA). Separation mobile phase A consisted of acetonitrile +0.1% formic acid, and separation mobile phase B consisted of water +0.1% formic acid. The injection volume was 5 μL, and the flow rate was 0.6 mL/min. The following linear gradients were used: 0 min 95% A and 1 min 95% A, 4 min 30% A and 5 min 30% A, 5.1 min 95% A, and 8 min 95% A.

The mass spectrometer was operated in full scan mode in the mass range from 50 to 1000 *m*/*z* and with an accumulation time of 250 ms. In ESI+ mode, the source temperature was set at 700 °C, the declustering potential at 30 V, the collision energy at 6 V, the ion spray voltage at 5120 V, the curtain gas at 25 psi, and ion source gases 1 and 2 at 60 psi. In ESI− mode, the source temperature was set at 650 °C, the declustering potential at −45 V, the collision energy at −6 V, the ion spray voltage at −3800 V, the curtain gas at 25 psi, and ion source gases 1 and 2 were set at 30 psi. The instrument was mass calibrated by automatic calibration after every two sample injections, infusing the Sciex Positive Calibration Solution (part no. 4460131, AB Sciex, Foster City, CA, USA) for positive mode and the Sciex Negative Calibration Solution (part no. 4460134, AB Sciex, Forster City, CA, USA) for negative mode. LC-MS system maintenance was performed at the beginning of each plate batch to ensure high-quality data. Moreover, to monitor the retention time, mass accuracy, and signal intensity, a system-suitability solution was injected. This solution was prepared in acetonitrile/water (50:50, *v*/*v*) and consisted of labeled amino acid standards at the following concentrations: alanine 13C315N (0.9 μg/mL), arginine 13C615N4 (1.8 μg/mL), glutamic acid 13C515N (1.5 μg/mL), methionine 13C515N (1.5 μg/mL), phenylalanine 13C915N (1.7 μg/mL), serine 13C315N (1.1 μg/mL), and valine 13C515N (1.2 μg/mL).

In addition, to equilibrate the LC-MS system before the sample ran, pooled QC samples were injected 15 times in both the positive and negative modes. At the end of the equilibration injection sequence, we injected blanks to avoid carryover. Samples were then analyzed in a randomized order, and pooled QC samples were injected between every eight samples.

### 2.6. Data Analysis

Profile-mode raw mass spectrometry data files were converted to the mzML format by ProteoWizard and subsequently centroided after applying a Savitzky–Golay filter using MSnbase [17]. All further data analysis was performed in R (version 4.2.0). Data sets were pre-processed (separately for both polarities and both data sets) using xcms (version 3.18.0). In brief, centWave [18] was used for chromatographic peak detection (parameters: ppm = 50, peakwidth = c(2, 20), snthresh = 10, mzdiff = 0.001, prefilter = c(3, 800), noise = 100, integrate = 2). The refineChromPeaks method was subsequently applied to merge neighboring or partially overlapping peaks (parameters: expandRt = 2.5, expandMz = 0.001, minProp = 0.75). After QC-sample-based alignment with the peak group method (parameters: minFraction = 0.95, extraPeaks = 50, span = 0.3, subsetAdjust = “average”). The peak density method was used for correspondence analysis (parameters: minFraction = 0.3, minSamples = 3, binSize = 0.015, bw = 2.3). Details on parameter choices, descriptions and the code of the full analyses are provided in R markdown files in the github repositories https://github.com/EuracBiomedicalResearch/VAMS_vs_intraveinous (accessed on 17 January 2023) and https://github.com/EuracBiomedicalResearch/mitra_short_term_stability (accessed on 17 January 2023). Missing peak intensities were filled in using the fillChromPeaks function from xcms (with the default settings from ChromPeakAreaParam).

For normalization, the first median scaling was applied to remove any global intensity differences between the samples, followed by a linear model-based within-batch normalization strategy similar to [19] to also adjust for signal drifts during the measurement runs. In detail, linear models describing the feature abundance as a function of the injection index were fitted to the intensities measured in QC samples (short-term stability data set) or in all samples (VAMS MS-workflow data set). Features for which the linear model was considered valid (i.e., based on a required minimum number of data points and spanning at least 3 quarters of the injection index range) were adjusted. For the VAMS MS workflow data set, an additional between-batch normalization was performed. The functions xcms:::rowFitModel and xcms:::applyModelAdjustment were used for this normalization. Further details can be found in the respective R markdown files mentioned above.

For the semi-targeted analysis, LC-MS features were annotated in each data set using the MetaboAnnotation [20] package: features’ *m/z* and retention time values were matched against those of pure standards previously measured on the same LC-MS setup. For the short-term stability data set, 58 and 76 features could be annotated with this method in positive and negative polarity for the VAMS MS-workflow data sets 56 and 77. For the untargeted analysis, the data sets were prefiltered keeping only features with a D-ratio < 0.5 [21] and with missing values (before gap-filling) in less than 50% (short-term stability data set) or 30% (VAMS MS-workflow data set) of the samples for at least one study group. This resulted in data sets with 8246 and 713 features for the short-term stability data set and the VAMS MS-workflow data set, respectively (10,060 and 998 features for negative polarity). Features (metabolites) were defined as present in a certain sample matrix if a chromatographic peak was detected in at least one-third of samples for that matrix. For the VAMS analysis, the abundances of the two available technical replicates were averaged for each feature to result in the abundance estimates used for the final differential abundance analysis. The linear model-based moderated t-test from Bioconductor’s limma package [22] was used for the differential abundance analysis, and the resulting *p*-values were adjusted for multiple hypothesis testing using the method from Benjamini and Hochberg. For the VAMS MS workflow data set, the linear model included the sample matrix and the participants’ sex as explanatory variables.

## 3. Results

### 3.1. Evaluation of Short-Term Stability

While the polar metabolome seems to be stable in VAMS devices if stored at −80 °C [10], the effects of short-term storage have not been studied extensively. To address this, we designed an experiment in which multiple VAMS samples were collected from a pooled human blood sample and stored at different conditions after letting them dry for 2 h (Figure 1A). 

The first set of VAMS samples was stored at room temperature for up to two weeks, and samples were extracted at different time points to evaluate changes in the metabolome over time (Figure 1A). The other four sets of samples were stored using bags with desiccants or were sealed under a vacuum and stored at room temperature or at 4 °C. Extracted samples were then stored at −80 °C, and all samples were analyzed together in one analytical batch at the end of the experiment.

The principal component analysis showed no evident clustering of the samples based on different storage conditions (Figure 1B). However, according to the first component, samples were clearly separated into three clusters depending on the time of storage (Figure 1B). Interestingly, regardless of the storage conditions, samples were stored for up to 6 h clusters together. A second cluster included all samples that were stored for 1 day. Among them, samples stored at 4 °C in bags with desiccants showed the highest distance to the samples stored for 2 and 6 h. The third cluster included all samples stored for periods longer than 1 day.

We then decided to evaluate how many features were significantly changed when comparing samples with different times of storage to the initial set of samples dried for 2 h at RT. The results, presented in Figure 1C, confirm the finding of the PCA analysis. Indeed, samples stored for 6 h showed very few changes with a variation lower than 3% except for the samples stored under a vacuum. The stability of the metabolome started to be affected from day 1, with a variation ranging from 10% up to 25% for samples stored in bags with desiccants at 4 °C. Finally, for samples stored for longer time periods, we observed variation in the metabolome ranging from 40% to 60%. After 1 week of storage, we noticed a decreased variation in the metabolome, but this may be attributed to no uniform changes in the metabolome among the samples, thereby resulting in higher variability within the replicates and among samples stored at the same conditions; therefore, lower number of significant features were detected. 

### 3.2. Validation of VAMS-MS-Based Metabolomics Workflow 

**Figure 2:**
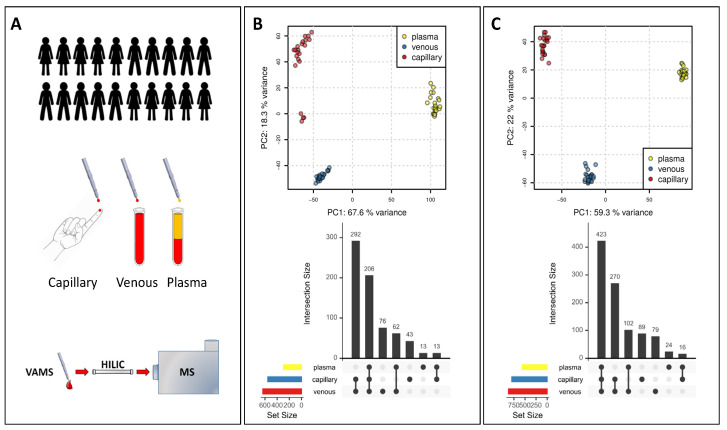
Validation of VAMS-LC-MS for metabolomics application. (**A**) Experimental design (**B**) PCA in positive ionization mode and comparison of metabolome coverage in different matrices. (**C**) PCA in negative ionization mode and comparison of metabolome coverage in different matrices.

We next aimed to determine the feasibility and usability of capillary blood collected using VAMS for metabolomics studies. To this end, we designed a study with 22 apparently healthy subjects (Figure 2A). From each participant, we collected capillary blood from the finger cup using two VAMS devices and venous blood using standard phlebotomy. From each phlebotomy collected EDTA tube, two VAMS devices were used to collect a venous whole blood sample. Finally, two VAMS devices were used to collect a plasma sample from the venous blood-derived plasma sample of each participant.

#### 3.2.1. Comparison of VAMS Samples from Different Matrices

We next compared the metabolome of capillary blood, venous blood, and plasma obtained by collecting VAMS samples from the study participants. A PCA analysis on the LC-MS feature abundances of the untargeted data set clearly showed three clusters both for positive and negative modes (Figure 2B,C). The PC1, which accounts for about 60% of the variance, clearly separates the plasma metabolome from the metabolome obtained in the blood. The PC2 components, accounting for about 20% of the variance, mainly separate capillary blood metabolome from venous blood metabolome (Figure 2B,C).

The clear difference between the plasma, venous, and capillary metabolomes highlighted by the PCA was confirmed by comparing features detected in all matrices or features selectively detected in one or two matrices only. From a qualitative point of view, the coverage of the metabolome is quite different. The highest number of features was obtained in venous blood, while, not unexpectedly, the lowest number was detected in the plasma in both the positive and negative modes (Figure 2B,C). In the positive mode (Figure 2B), 206 features were detected in all three matrices, while 76 features were detected only in venous blood, 43 features were found only in capillary blood, and 13 features only in plasma. The largest overlap of detected features was between venous and capillary samples (292), followed by venous and plasma samples (62). The smallest overlap was between capillary and plasma samples (13 features).

In the negative mode (Figure 2B), 423 features were detected in all three matrices, while 79 features were detected only in venous blood, 89 only in capillary blood, and 24 only in plasma. The largest overlap of detected features was again between venous and capillary blood samples (270 features), followed by capillary blood and plasma samples (102 features). Similar to positive polarity and also for negative polarity, capillary blood and plasma samples showed the lowest overlap of detected features (16 features).

**Figure 3:**
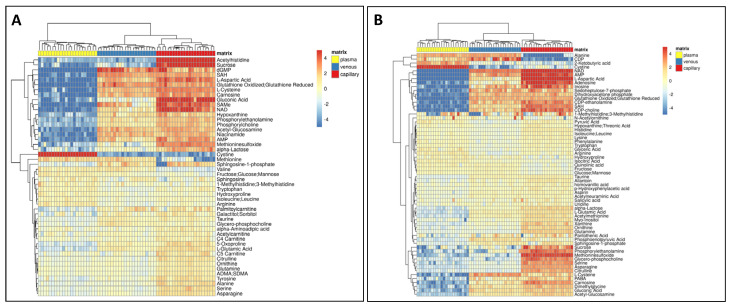
Quantitative comparison of the metabolome among different matrices. (**A**) Heatmap of metabolites significantly changing in positive mode. (**B**) In negative mode.

The difference among matrices is not only qualitative regarding the coverage of the metabolome, but also quantitative, as shown in the heat map in Figure 3. We found metabolites that were detected only in capillary and venous blood, such as intracellular metabolites but we also noted that metabolites detected in all matrices had considerably different abundances reflecting differences in metabolite concentrations between the matrices. 

**Figure 4:**
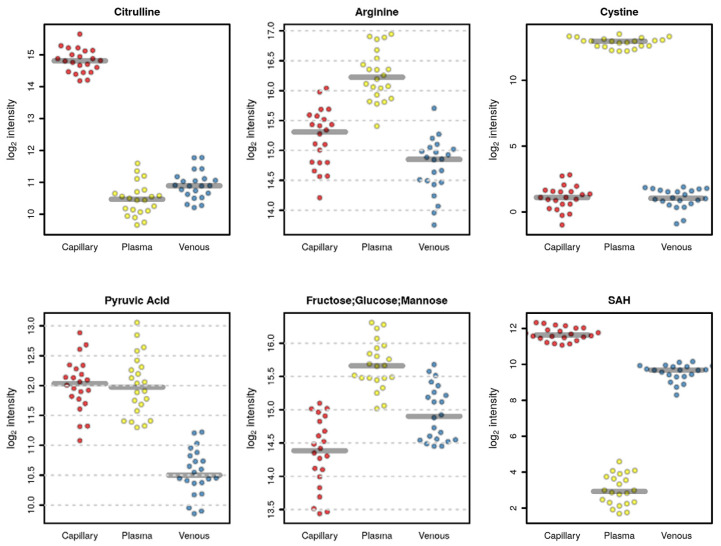
Plots of selected metabolites that are significantly different among the three matrices (red, capillary; yellow, plasma; blue, venous blood).

For instance, some metabolites such as S-adenosylhomocysteine (SAH) (Figure 4) and phosphorylcholine and glutathione (data not shown) were mainly detected in venous and capillary blood. Several amino acids showed higher values in plasma, such as cystine and arginine, with some exceptions, such as citrulline which was detected at higher levels in the capillary blood, while valine had a lower abundance in capillary blood (Figure 4). As expected, glucose had higher levels in plasma samples since, inside the cells, it was expected to be rapidly metabolized through glycolysis (Figure 4).

**Figure 5:**
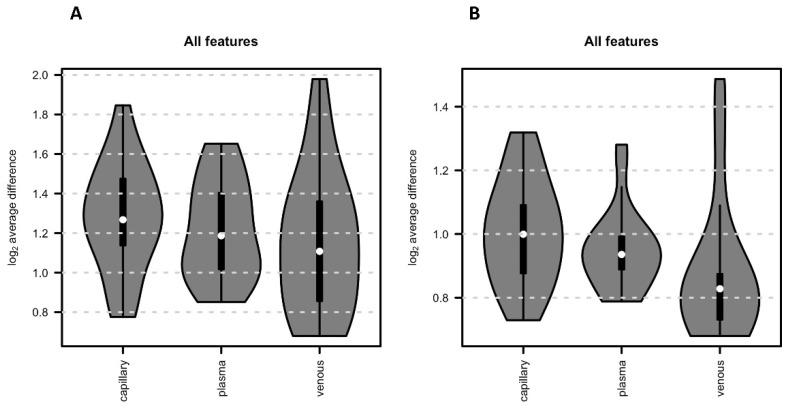
Reproducibility. The average absolute difference of intensities for each replicate pair (i.e., metabolic profile from the 2 VAMS devices per sample) across the 22 VAMS sample replicate pairs. (**A**) All features obtained in positive mode. (**B**) All features obtained in negative mode.

Regarding reproducibility, from the PCA (Figure 2B,C), it seemed that venous samples had the lowest variance between the replicates (i.e., participants’ samples for the same sample matrix), while a larger variation was visible for capillary samples. To evaluate this further, we calculated for each feature the difference in abundance between the two replicated VAMS devices for each participant in each sample matrix. These two VAMS devices per sample represent technical replicates and allow, thus, to evaluate technical differences in the sample collection. Indeed, the average difference of abundances across all features was lowest for venous samples and highest for capillary samples for both positive as well as negative polarity (see Figure 5).

#### 3.2.2. Gender Polar Metabolome of VAMS Samples from Different Matrices

**Figure 6:**
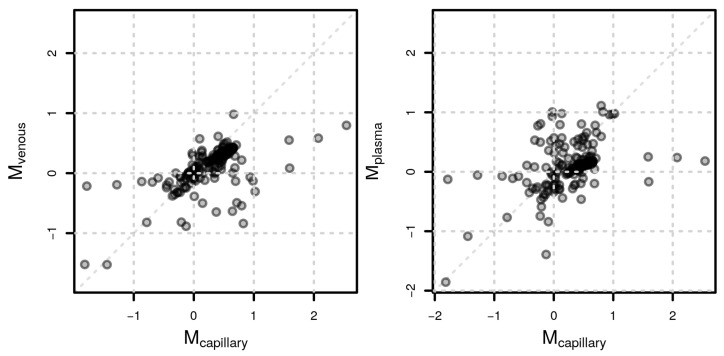
Sex-related metabolite differences. Comparison of log2 fold change values (M) representing differences in feature abundances between male and female participants between capillary and venous samples (**left**) and capillary and plasma samples (**right**) for positive polarity.

To evaluate whether VAMS capillary samples would lead to similar results as a plasma or venous blood samples, we aimed to compare feature abundances between male and female study participants for each sample matrix. To ensure comparability, we restricted the analysis to features that were presented in each of the three sample matrices reducing the untargeted data set to between 206 and 418 features for positive and negative polarity, respectively. The correlation of log2 fold change values representing the abundance differences was high between the sample matrices (Spearman’s rho of 0.66 between capillary and venous, and 0.53 between capillary and plasma samples in positive polarity and 0.61 and 0.42 in negative polarity; Figure 6). 

Very few annotated metabolites changed significantly after adjusting for multiple hypothesis testing. Among the significant metabolites was gluconic acid, an intracellular metabolite [23] that showed significantly higher concentrations in male samples both in capillary and venous blood but not in the plasma due to its very low concentration in plasma (Figure 6). Other metabolites such as citrulline, aspartic acid, and SAH all had a raw *p*-value lower than 0.05, which was, however, no longer significant after adjusting for multiple hypothesis testing. Importantly, however, the trends were similar for all metabolites, with some exceptions for metabolites detected at very low levels in one matrix, such as aspartic acid in the plasma.

## 4. Discussion

Volumetric absorptive microsampling (VAMS) is a recently developed sample collection technology that allows single-drop blood collection in a minimally invasive manner. Blood biomolecules can then be extracted and processed for analysis using several analytical platforms. Microsampling has become a widely used procedure, especially for therapeutic drug monitoring (TDM) [24,25,26]. Moreover, the VAMS device is a good candidate for blood microsampling in a standardized way with a less invasive procedure, enabling both more frequent sampling and remote collection. In addition, the minimal volume of the sample collected onto VAMS enables longitudinal metabolomic studies and is particularly in line with the 3R’s principle [14]. The integration of VAMS with conventional mass spectrometry (MS)-based metabolomics approaches is an attractive solution for different applications, ranging from animal studies to human population studies [27,28] and longitudinal studies [29]. 

However, to implement this procedure for metabolomics studies, there are still some open questions. One of them is the stability of the metabolome of blood samples collected with VAMS. Based on our previous work, VAMS samples collected for metabolomics should be stored at −80 °C after the sampling to prevent changes in the overall metabolome profile for longer storage periods [10]. 

In this study, we focused first on the short-term stability of the metabolome collected by VAMS by comparing different storage conditions. Long-term storage at room temperature is not possible due to the poor stability of the blood metabolome once collected by VAMS devices [10]. We thus tested ways to potentially improve the short-term storage at room temperature and evaluated how long samples could be kept at room temperature before needing to be placed at −80 °C. Short-time storage solutions not requiring deep freezing would be key for remote sampling strategies. Storing samples at −80 °C might be difficult for some clinical and research applications, so the goal of this experiment was to identify the maximum storage time at more accessible temperatures at which the metabolome would remain preserved. To this end, we thus evaluated various storage conditions, including closed bags containing desiccants, closed bags sealed under a vacuum, and different storage temperatures.

Unfortunately, our results demonstrated that VAMS samples could be kept at room temperature only for up to 6 h in all tested conditions. After 6 h, the polar metabolome started to change significantly (Figure 1), precluding the use of VAMS for remote sampling unless the samples could be placed at −80 °C within the first 6 h after collection. These results are, however, based on the untargeted metabolomics approach for detecting the polar metabolome and other metabolites or lipids that might be stable for a longer time span in VAMS devices.

Combining experiments for the short-term storage presented in this manuscript with the data related to the long-term storage and sample processing presented before by our group [10], we now have a more complete knowledge of the potential of the VAMS samples for metabolomics. 

Most metabolomics studies use plasma or serum samples [30], while the analysis of whole blood is less common. However, in the clinical setting, whole blood is commonly used to investigate molecular biomarkers that are important for patient diagnosis, prognosis, and monitoring [31,32]. The usual method for whole blood collection is venous phlebotomy; in addition, blood can be also collected as capillary blood [2,33]. Regarding the metabolomic profile, capillary blood can show a different metabolome compared to the one obtained from venous blood. To evaluate the feasibility of capillary blood as a matrix for metabolomics studies, we designed and conducted a specific study. We recruited 22 healthy participants and collected from each capillary blood, venous blood, and plasma. All samples were collected using VAMS devices, even the phlebotomy-based venous blood sample, to reduce sampling effects. 

We conducted a qualitative and quantitative comparison of the polar metabolome obtained from each matrix. As expected, the whole blood resulted in a higher number of detected features (Figure 2), thus providing a potentially higher coverage of the metabolome, including also intracellular metabolites coming from the blood cells, in particular, red blood cells [34,35,36]. The lowest number of features was detected in the plasma; nonetheless, some metabolites, such as cystine and glucose, were detected at higher levels in plasma (Figure 3 and Figure 4), suggesting that these metabolites are metabolized faster or more within the cells resulting in lower concentrations inside blood cells [37].

Interestingly, the metabolomes obtained from capillary blood and venous blood also differed both qualitatively as well as quantitatively. This might be due to a different composition of blood cells present in venous and capillary blood but also to a different composition of circulating metabolites.

We also checked the reproducibility of the three different investigated matrices. Each sample was collected twice, with two separate VAMS devices, and we compared the average absolute difference of intensities for each replicate pair across the 22 VAMS sample replicate pairs. Capillary samples showed higher variability in this analysis compared to venous and plasma samples (Figure 2A and Figure 5). The capillary samples were, however, collected from two different blood drops, while both venous blood and plasma were collected from the same EDTA tube. Therefore, while venous blood and plasma blood can be considered technical replicates, capillary VAMS are two consecutive samples obtained from two different blood samples, which might explain the observed higher variability.

Despite the higher variability in capillary blood, the three matrices provided similar results comparing feature abundances between female and male participants. Indeed, correlation coefficients were high, as shown in Figure 6, suggesting that the usage of VAMS samples in metabolomics experiments, independently of the sample matrix employed, can capture biological differences of interest. Finally, we compared single metabolites that changed in each matrix due to sex differences. Unfortunately, due to the high variability in the data set combined with the low sample size, for very few annotated metabolites, the observed abundances were significant after adjusting for multiple hypothesis testing. For instance, male participants had significantly higher levels of gluconic acid both in the capillary and venous blood samples but not in plasma (Figure 6). Gluconic acid is a common food and drug constituent, and we previously demonstrated that it can also be derived by glucose oxidation and further phosphorylated to be used in the pentose phosphate pathway [23,38].

**Figure 7:**
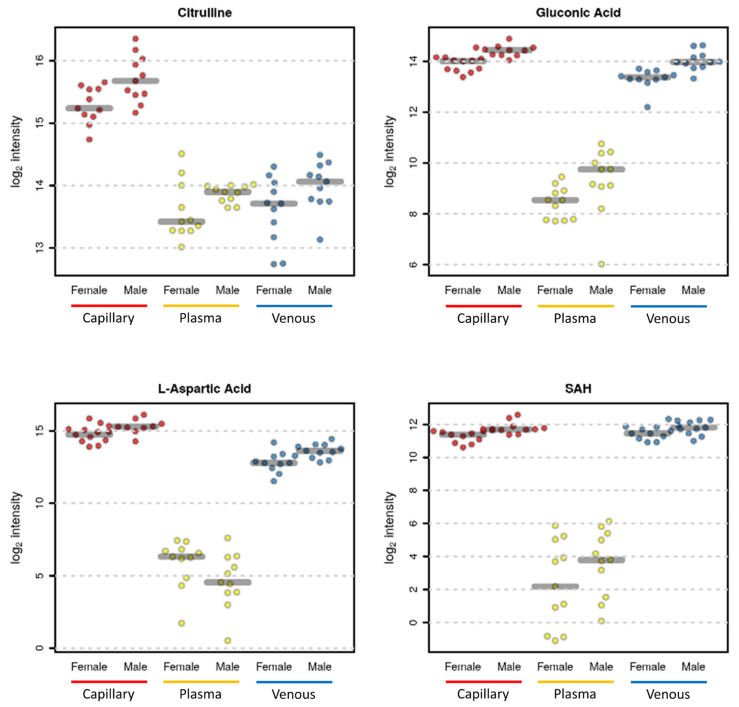
Selected sex-related metabolite differences. Citrulline in capillary blood (*p* value = 0.009—adjusted *p* value = 0.118), in plasma (*p* value = 0.082—adjusted *p* value = 0.773), and in venous blood (*p* value = 0.060—adjusted *p* value = 0.387). Gluconic Acid in capillary blood (*p* value = 0.00005—adjusted *p* value = 0.004), in plasma (*p* value = 0.046—adjusted *p* value = 0.322), and in venous blood (*p* value = 0.0003—adjusted *p* value = 0.021). Aspartic acid in capillary blood (*p* value = 0.009—adjusted *p* value = 0.118), in plasma (*p* value = 0.110—adjusted *p* value = 0.773), and in venous blood (*p* value = 0.006—adjusted *p* value = 0.215). S-adenosyl homocysteine (SAH) in capillary blood (*p* value = 0.002—adjusted *p* value = 0.118), in plasma (*p* value = 0.170—adjusted *p* value = 0.782), and in venous blood (*p* value = 0.055—adjusted *p* value = 0.387).

Several other metabolites, although not significant after adjusting for multiple hypothesis testing, had a raw *p*-value lower than 0.05 (Figure 7). The trend of most of the metabolites was similar in all matrices (Figure 7), demonstrating that the information captured by the capillary blood metabolome is similar to those of both the plasma and venous blood metabolomes. Moreover, the capillary blood metabolome offers the possibility of investigating a larger number of metabolites since it provides a higher coverage due to the presence of both intracellular metabolites and plasma metabolites. For instance, aspartic acid is an amino acid usually found at very low concentrations in the plasma [30], while it is present at higher concentrations in erythrocytes [37].

According to our findings, the use of VAMS for remote sampling in metabolomics studies might suffer from higher variability; however, it still represents a valid and interesting sample collection procedure. This is especially true if frequent blood samplings are required, in the case of longitudinal studies and/or in studies where there is a need for social distancing to avoid unnecessary personal contact. Further studies aimed to improve the standardization procedure of VAMS sampling, handling, and storage should follow. 

## 5. Conclusions

In our previous work, we proved the long-term stability of the polar metabolome in blood samples collected using VAMS devices if stored immediately after drying for 2 h at room temperature, at −80 °C. 

In this study, we evaluated the possibility of improving the short-term stability of the blood metabolome collected by VAMS. We tried to minimize the effect of oxidation and humidity using sealed bags with desiccants or sealed vacuum bags to enable the storage of VAMS samples for a longer time at room temperature or at 4 °C without major changes in the metabolome.

Our results indicate that significant changes in the quantitative polar metabolome composition already began after 6 h, excluding the possibility of the use of VAMS for metabolomics studies using remote sampling unless both storage and transport are performed under refrigeration at −80 °C.

To further prove the feasibility of VAMS collection for metabolomics, we compared capillary blood collected from the finger with venous blood and plasma obtained from 22 healthy participants.

We found capillary blood to provide better coverage of the metabolome, which also includes intracellular metabolites from blood cells. In addition, the polar metabolome from VAMS-based capillary samples was equally able to capture differences between male and female study participants than the polar metabolome from venous or plasma samples.

In summary, while the low stability at room temperature precludes a direct use of VAMS devices for untargeted metabolomics studies using a remote sampling strategy, its minimal sample volume and the minimal invasiveness make it a powerful tool for metabolomics studies using animal models or requiring frequent blood sample collection to study dynamic metabolite changes.

## Data Availability

Not applicable.
